# Chronic activation of heme free soluble guanylate cyclase leads to cardio-renal protection in experimental hypertension

**DOI:** 10.1186/2050-6511-14-S1-P30

**Published:** 2013-08-29

**Authors:** Linda S Hoffmann, Axel Kretschmer, Bettina Lawrenz, Andreas Hucke, Berthold Hocher, Johannes-Peter Stasch

**Affiliations:** 1 Pharma Research Centre, Bayer HealthCare, Aprather Weg 18a, Wuppertal, Germany; 2 Martin-Luther-University, School of Pharmacy, Wolfgang-Langenbeck-Str.4,Halle,Germany; 3 Current address, Institute of Pharmacology and Toxicology, University of Bonn, Sigmund-Freud- Strasse 25, 53127 Bonn, Germany; 4 Center for Cardiovascular Research, Department of Pharmacology and Toxicology, Charité, Campus Mitte, Hessische Str. 3-4, Berlin, Germany

## Background

The cytoprotective NO/sGC/cGMP-signalling pathway is impaired under oxidative stress conditions as observed for cardiovascular diseases due to oxidation and subsequent loss of the prosthetic sGC heme group [1,2]. Thus, the pool of heme free sGC is increased under pathological conditions. The unique property of sGC activators such as cinaciguat (BAY 58—2667) to selectively activate heme free sGC allows to target the disease associated form of sGC. In this study a therapeutic effect of long-term activation of heme free sGC was investigated in an experimental model of salt sensitive hypertension.

## Material and methods

Male Dahl/ss rats, 7 weeks of age, were randomly allocated into two groups: control group (n=20) receiving a high salt diet containing 8 % NaCl, cinaciguat group (n=17) receiving 1000 ppm cinaciguat in the high salt diet. 6 h urine samples were collected at week 19. Systolic blood pressure and heart rate were measured using tail-cuff at week 0, 6, 9, 12, 15 and 21. High resolution echocardiography was performed at week 20. The study was terminated at week 21 (50 % survival of the control group). Plasma and urine parameters were analyzed using a Multichannel Autoanalyzer. ANP and cGMP were analysed using radioimmunoassay kits. Plasma and urine concentrations of osteopontin (OPN) were determined using immunoassay kits. Gene expression of biomarkers was analysed by qRT-PCR.

## Results

Cinaciguat highly significantly improved survival (Χ^2^=8) compared to control. Increase in blood pressure and heart rate was significantly lower in cinaciguat treated rats than in control rats. M-mode echocardiography showed increased fractional shortening and a higher diameter increase of the septum between systole and diastole. Urine concentrations of urea and protein and plasma levels of uric acid were reduced in cinaciguat compared to control. Cinaciguat prevented the increase in expression of pro-fibrotic and pro-inflammatory biomarkers in kidney and left ventricle compared to control. Histopathological examination of organs from deceased animals revealed that especially fibrotic and inflammatory events in the heart and kidney were the cause of death. These pathological lesions were markedly decreased in cinaciguat treated animals.

## Conclusion

The data clearly shows that long-term activation of heme free sGC with cinaciguat leads to cardio-renal protection demonstrated by reduced mortality, improved cardiac and renal function, lower increase in blood pressure and reduced inflammatory and fibrotic events. These results underline the promising potential of sGC activators to treat cardiovascular diseases by targeting the heme free form of sGC.
